# Cognitive Burden in Emergency Care: A Qualitative Exploratory Study of Emergency Nurses

**DOI:** 10.1155/jonm/6332434

**Published:** 2025-11-30

**Authors:** Anu Surendran, Lisa Beccaria, Peter McIlveen

**Affiliations:** ^1^School of Nursing and Midwifery, University of Southern Queensland, Toowoomba, Queensland, Australia; ^2^School of Education, University of Southern Queensland, Toowoomba, Queensland, Australia

**Keywords:** cognitive mental workload, emergency nurse, nurse, qualitative study, reflexive thematic analysis

## Abstract

**Aim:**

This study explores emergency nurses' lived experiences of cognitive mental workload (CMW) within high-pressure, resource-limited environments, where excessive mental demands may compromise both clinician well-being and care delivery.

**Method:**

An exploratory qualitative research design was used with registered nurses (*n* = 10) working in Australian rural emergency departments. Online semistructured interviews were conducted in 2023. The collected data were analysed using reflective thematic analysis.

**Results:**

The data analysis revealed four primary contributors of high CMW among nurses during care delivery, namely, external demands, excessive cognitive processing, emotional demands and individual characteristics. Despite challenges in describing the phenomenon, participants clearly associated their high CMW to adverse patient outcomes like patient harm and near misses as well as negative impacts on their own welfare, such as burnout, compassion fatigue and intention to leave profession.

**Conclusion:**

This study offers new perspectives on emergency nurses' CMW experiences, foregrounding their lived experience in high pressure, emotionally charged and dynamic clinical settings. By giving voices to the often overlooked phenomenon in nursing, it has revealed the critical impact of the health professional's CMW on their well-being, patient safety and care quality.

**Implications for the Profession and Patient Care:**

In depth knowledge about the phenomenon can inform tailored measurement tools and targeted strategies for nurses, to reduce errors and enhance care delivery.

**Impact:**

Policymakers can integrate the insights to strengthen healthcare infrastructure and policies that reduce error-related patient harm and support nurse well-being for reducing resource and financial expenditure.

## 1. Introduction

Nurses' cognitive mental workload (CMW) during urgent care in resource constricted, fast paced and dynamic environment of emergency department (ED) poses threat to patient care. Grounded in Sweller's cognitive load theory (1988), CMW occurs when cognitive demands exceed brain's limited cognitive capacity. Globally, EDs are overcrowded, complex and uncertain environments with limited physical resources, time pressures, work complexity and intensity and constantly looming crises [1, 2]. This environment imposes high cognitive demands [3] and emotional demands [4] on emergency nurses who must deploy multiple strategies [5] that require considerable cognitive resources from a finite reserve [6]. The imbalance between high cognitive demand and cognitive resources availability in their cognitive architecture contributes to the emergency nurses' CMW experience [3, 7]. Their high CMW can contribute to delay, omission or substandard care task execution precipitating patient harm, substandard care delivery and the emotional burden on the nurses leading to employee burnout resulting in extensive financial impact on healthcare sector [2]. Despite the impact of CMW, there is limited research into emergency nurses' lived CMW experiences. This study addresses the knowledge gap by exploring emergency nurses' CMW experiences during care delivery.

## 2. Background

ED overcrowding threatens patient safety, compromises care quality and strains healthcare workforce, especially nurses [5]. The nurses must multitask and prioritise while experiencing interruptions, distractions and disruptions under time pressures [1, 8]. These work conditions generate significant cognitive workload against limited resources [9, 10]. Yuan et al.'s [11] systematic review and meta-analysis study reported a variation of mental workload among nurses working in different areas, with emergency nurses having the highest mental workload. Despite the potential risks of high CMW [3, 12] and emerging interest in the field [1, 13], there are very few credible and effective optimisation strategies to reduce and mitigate the adverse effects of high CMW during emergency nursing care [2].

Emerging research has investigated the CMW of emergency nurses, possibly driven in part by heightened awareness of workforce strain and well-being in the post-COVID-19 context. Apart from empirically proving that 90% experienced moderately high mental workload during their work in ED, Hao et al.'s [13] cross-sectional latent profile analysis classified 305 emergency nurses into three distinct mental workload profiles based on their subjective mental workload score and their individual characteristics. Park et al. [1] quantified the mental workload of emergency nurses' individual tasks and established that cardiopulmonary resuscitation (CPR) caused the highest mental workload, followed by family interruptions. Both studies used the National Aeronautics and Space Administration Task Load Index (NASA-TLX) to subjectively measure the phenomenon due to the lack of a customised tool for emergency nurses. Similar to Hao et al. [13], the multisite study of Soto-Castellón et al. [3] found a direct connection between environmental and individual factors on nurses' CMW. Kim et al. [7] found that interruptions in a laboratory setting significantly increased emergency nurses' mental workload, compromising patient safety and performance. Forsyth et al. [8] used subjective and objective measures to show that nurses were interrupted every six to 7 min, substantially increasing their workload. [14] identified a negative correlation between nurses' mental workload and patient satisfaction and outcomes, noting that high mental workload impaired cognitive abilities, increasing risk of human error leading to adverse patient outcomes and substandard care.

The nomenclature of the phenomenon is confusing due to inherent complexity and intricacies. Various labels, such as cognitive load, cognitive workload, mental load and mental workload, are seemingly utilised in the literature to represent similar phenomena [10]. As defining and distinguishing the difference between the terms is beyond the scope of the study, an umbrella term ‘CMW' has been adapted for the present study, representing the mental effort required to perform task and the imbalance that arises between cognitive demand and cognitive resources in the process [2].

Cognitive science theories offer potential conceptualisations of CMW. Sweller's [6] cognitive load theory stipulates that individuals experience cognitive load when cognitive demands exceed mental capacity or working memory capacity. According to the theory, time pressures, task complexity or work intensity impose cognitive load, which is managed by finite cognitive resources available in their cognitive architecture [6]. Thus, the finite pool of cognitive resources could be depleted for managing cognitive demands (i.e., mental effort to perform cognitive and physical tasks) and emotional demands (i.e., mental effort to manage emotions) [10]. High mental workload is experienced when the limited cognitive resources get depleted at a rate faster than the rate at which it is replenished in human cognitive architecture, resulting in scarcity of resources to manage cognitive demand [10]. Wickens' [15] multiple resource theory explains how human brain utilises multiple, distinct and finite pools of cognitive resources for various cognitive operations such as attention, information processing, thinking, decision making and emotional management. The theory suggests that performance is optimised when tasks draw on different pools of limited cognitive resources but degrades when tasks compete for similar pool of resources, which results in errors, failures or suboptimal performance, due to cognitive overload. Therefore, the high error rates in ED [16] could be attributed to emergency nurses' high workload.

Additionally, when CMW level exceeds individual endurance, nurses face exhaustion and burnout, leading to intention to leave [17]. Beyond the phenomenological within-person experience of CMW, Longo et al. [9] and Van Acker et al. [10] argue that both circumstance and environment influence the specific nature of CMW and are only accessible through an individual's subjective expression or disclosure. Further, Guastello et al. [18] found that multiple individual traits and abilities like coping abilities, psychosocial state and work ethics play a crucial part in enduring CMW. Therefore, task characteristics, environment, individual and personal traits affect an individual's CMW perceptions.

Nonetheless, inconclusiveness about the current understanding of CMW requires further investigation in general [9, 10]. Moreover, rarely any lived experience studies have been conducted among nurses to explore and gain understanding of the complex and multifaceted phenomenon. Understanding CMW is crucial for establishing quality and safety in emergency healthcare delivery and safeguarding nurses' well-being and this study addresses the gap.

## 3. The Study

### 3.1. Aim

The present research is an exploratory enquiry aimed at enhancing understanding of emergency nurses' CMW through explorations of their lived experience of the phenomenon. The research question was: What are the subjective experiences of emergency nurses' CMW during patient care? What are the optimum CMW levels for emergency nurses to maintain high performance and ensure patient safety?

## 4. Methodology

### 4.1. Research Design

Reflexive thematic analysis (RTA) [19] was used to explore nurses' reports of their subjective experiences. The study was reported according to Braun and Clarke's [19] the Big Q Qualitative Research Guideline (BQQRG). BQQRG facilitated flexibility and contextual meaning-making as well as valued subjectivity and reflexivity which cannot be achieved through traditional reporting checklists, due to its rigidity [19].

### 4.2. Ethical Considerations

The study protocol of the qualitative inquiry was initially published in Open Science Framework (https://doi.org/10.17605/OSF.IO/3UMSH). The study received ethics approval in April 2023, from the Human Research Ethics Committee of University of Southern Queensland (approval number: H22REA276). The research is conducted in accordance with National Health and Medical Research Council's Australian National Statement on Ethical Conduct in Human Research.

### 4.3. Participant Recruitment

A homogenous purposive sample of 10 registered nurses (RNs) working in rural EDs in Australia participated in the study. Their years of experience in emergency nursing varied between four to sixteen years. To achieve epistemological alignment with RTA, *information power* [20] was used to decide the sample size based on rural nurses' likelihood of experiencing high CMW because of workplace demands [21]. Information power was achieved with 10 interviews that provided rich and relevant data to address the research question and support meaningful analysis. Using information power instead of thematic saturation facilitated interpretivist paradigm alignment, which emphasised depth, meaning and subjective richness of individual perspectives over quantification and generalisability.

Recruitment involved the newsletter of ‘CRANA Plus' with nationwide reach and advertising on private social media sites of Australian rural emergency nurses (RENs) found by searching. Participants were required to hold Australian health professional registration as RNs and to have worked more than 3 months in a rural ED. RNs employed by labour hire agencies were excluded from this study due to follow-up difficulties.

Despite the nationwide invitation advertisement, a few participants (*n* = 7) demonstrated interest in the study. Of the seven, four participants were consented and fully participated in the study. Others (*n* = 3) who expressed and gave their consent to participate in the study did not respond to our email invitation to book online interview. Though the reasons for nonparticipation and limited attrition (*n* = 3) are unknown, to achieve adequate information power and data richness, we resorted to snowball sampling. The initially recruited participants (*n* = 4) were encouraged to share details of our study in their social media and advocate our study among their professional contacts. The step resulted in the remaining participant recruitment (*n* = 6). However, the use of social media and snowball sampling resulted in a few participants (*n* = 3), having preexisting professional acquaintance with AS. Neither supervisory hierarchy nor power dynamics were involved; rather it was a collegial relationship. Nevertheless, potential bias was actively prevented through rigorous bracketing (viz. interview the interviewer and journal keeping) and strict adherence to interview protocol to uphold data trustworthiness.

### 4.4. Data Collection and Study Settings

One hour long online semistructured interviews were conducted once for each participant by AS using a peer-reviewed interview guide. AS's minimal qualitative research experience as a doctoral candidate was compensated by the guidance, supervision and expertise of PM and LB. No personal data other than years of experience was collected during the interview to maintain privacy. Sample prompts included, ‘Tell me about your experience of cognitive workload at work, How do you explain that experience?, How do you make decisions during high CMW state?, What extra steps do you take to ensure your well-being? and How do you control your emotions in highly stressful situations?'. The interview guide was initially tested by the interviewer with another rural nurse who did not participate in the study for validity. Interviews that occurred between June 2023 and August 2023 were digitally recorded. Due to the online nature of the interview, the research team had no control over interview settings. However, the invitation to participate recommended to participants that interviews were to be conducted in a calm and private space, away from work and other life commitments, to reduce distraction and ensure privacy.

### 4.5. Data Transcription and Management

Audio recording was digitally transcribed concurrently to the audio recording, utilising the inbuilt feature in the online meeting platform, and AS performed correction of the transcript. All participants were deidentified as a REN with a serial number ranging from one to ten according to the order in which they were interviewed. The interview recordings were transcribed electronically and then cross-checked with the audio recording. Participants were offered the opportunity to request their transcripts. However, no requests were received. The corrected verbatim transcripts were entered into NVivo. Deidentified data were stored for analysis and shared with the research team. As part of their invitation to participate, participants were advised that the data would be stored and later destroyed according to the ethical approval.

### 4.6. Data Analysis

Data analysis used the six-phase process of RTA [22] and is elaborately listed with justification in Table 1. Given the exploratory nature of the study, no predetermined code books were used. Due to the multifaceted nature and complexity of the implicit phenomenon, equal importance was given to both semantic and latent meaning in each line of the transcripts. AS manually coded the simplest units of data from each line (inline coding) and then discussed with coauthors PM and LB to ensure appropriateness and transparency. During theme generation, patterns were identified, and codes were organised based on a specific commonality, informed by theory. Defining, demarcating and refining themes involved an iterative and recursive process of deep engagement with data; thematic mapping to visualise coherence of various subthemes and member checking to eschew over-assumptions. Theme names were carefully curated to entail the essence and scope of each theme. Use of NVivo for data analysis and management provided a structured and traceable analytical process. Its inbuilt memo and annotation facility allowed documentation of analytical decisions and ongoing reflexivity, which improved transparency, credibility and rigour of this analysis. Rockmann and Vough's [23] protocol guided the selection of quotes and writing through storyboarding and composing effectively to include participants' and authors' voices.

### 4.7. Rigour and Reflexivity

The principal investigator, AS, is an emergency nurse with firsthand experience of CMW and performed the role as an *insider* who acted as an interpreter of participants' statements about their experiences. The direct experience with the phenomenon and professional similarity enabled rich access to nuanced data and facilitated trust during the interview. Awareness of over-familiarity and tendency to shape interpretations by AS was mitigated by implementing multiple strategies. *Interviewing the interviewer* was carried out prior to the participant interviews, to explicate AS's standing and reinstate self-awareness [24]. Reflective journalling was undertaken during data collection and analysis to explore and elicit assumptions, values and positionality. Weekly meetings between AS and coinvestigators PM and LB interrogated this *insider* perspective to ensure interpretations were meaningful and defensible considering the data. These conversations included the interviewers' reflections on reflexive journal notes, justifications of data interpretation, code and theme organisation in NVivo, and conceptualisations of codes and themes as a process of theory triangulation by Morgan [25]. Most steps performed to ensure rigour, transparency and trustworthiness are detailed in Table 1 along with RTA steps.

## 5. Findings

**Figure 1:**
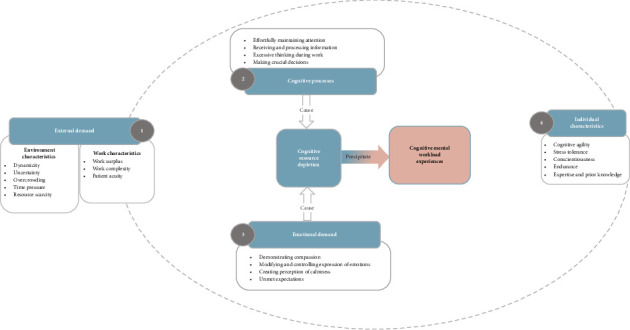
Thematic representation of emergency nurses' CMW experience.

The key findings were derived from nurses' CMW experiences related to their sensory processing, attention, information processing, working memory utilisation, thinking, decision making, problem solving, selection and execution of clinical tasks and their emotions during their work. Participants described demands emanating from both internal and external sources. Internal demands came from high volumes of sensory inputs, excessive information processing and multiple concurrent cognitive and memory processing activities. External demands included their challenging work environment (e.g., uncertainty, overcrowding, time pressure and resource scarcity) and work characteristics (e.g., surplus tasks, work complexity and patient acuity). Participants' individual characteristics affected their management of CMW.  Figure 1 presents the elements of CMW (e.g., external demands, cognitive processes, emotional demands and individual characteristics).

### 5.1. External Demand

External demand refers to an individual's task characteristics and environmental factors that are outside the individual. These are external factors that demand mental effort to process, respond and manage a stimulus, task or multiple actions at a point in time. Multiple features of the ED work environment were the substrate of CMW. The participants used expressions such as chaotic, crazy and out of control to describe their **disrupted workflow and the sheer volume** of presentations at peak times. REN 7 described the chaos as ‘*being unable to concentrate just on one decision and not five or six other decisions that have to be made at the same time*'. Often due to the chaos, they felt ‘*like a ping pong ball…, in those machines going Ting-Ting-Ting, from one thing to another*' (REN 2). Participants described **sudden and unexpected increases** in work intensity, complexity and acuity. ‘*Activity will increase three or four-fold from the baseline*' (REN 10), *and* ‘*you never know what's going to come through the doors… like, a complete mental 180 degree*' (REN 8).

In addition to their personal workloads, participants took on additional responsibilities. REN 1 said, ‘*I am the major problem solver in the department. That can be quite a huge mental load on top of own patients*' (REN 1). REN 6 described rapidly shifting work tasks, ‘*I've gone from being triage nurse, to resus nurse, to team leader—all in one. And there was sort of no one to call for help*'. This chaos evoked **anxiety and fear of making mistakes**. ‘*I really feel stressed coming home because I worry …there is something that I could have missed because of the rapidity of providing care*' (REN 1). ‘*When you've got just too…many balls in the air to juggle. If one of them is going to fall;* that is *going to be a chain effect. Then all the balls are going to fall off*' (REN 8).

**Resource deficits** were evident in admitting and treating patients, particularly when there was a shortage of beds. Similarly, equipment shortages inducing workload were evident, ‘*We didn't have all equipment in the department*. *So, it ended up on my shoulders to gather it from around the hospital. For that I had to leave the department and continuously run up and down to gather it*' (REN 1).

Like equipment shortages, the **availability of critical skillsets** among clinicians was a concern. ‘*I am working with junior nurses in major areas like resuscitation room and triage area. You're going to be thinking about their patients' safety while meeting your own patients' needs. Not because they're bad nurses, but just because they don't have that experience behind them*' (REN 4).

The nature of patient presentations triggering a **sense of urgency and time pressure** was associated with not being sufficiently caring: ‘*we're not able to spend a lot of time with our patient because we simply can't*' (REN 7). Furthermore, ‘*errors happen when people are rushed to do things and are not allowed enough time to go through it methodically*' (REN 7). ‘*I feel like I'm being squeezed*' (REN 2).

### 5.2. Cognitive Processes

Cognitive processes are the internal mental operations through which individuals perceive, attend to, interpret, store, retrieve and respond to information. For all participants, it was the performing of versatile complex cognitive tasks to provide care, which was like ‘*my brain constantly going 1,00,000 miles an hour*' (REN 4).

**Effortfully maintaining attention** amid competing stimuli demands attention, such that ‘*you're constantly having to juggle and reprioritize*' (REN 3). The uneasiness of sustaining attention concurrently to multiple stimuli was a widespread emotion among participants. Sustaining attention is burdensome, ‘*when you're in resus [patient resuscitation] it is the concentration, you burn… And you don't even know how much mental energy it takes*. *And you realise it after the resus is over that how much it took from you. Usually, you see it immediately on the faces of your colleagues*' (REN 1). Emotions are evoked by the mental energy invested in staying vigilant, detecting patient deteriorations and preventing errors effortfully.

**Relentless distraction and interruptions** emerged as hindrance to conserving attention and concentrating on patients, tasks or thoughts while working. ‘*It definitely takes its toll because it breaks your focus, and you lose track of where you were with patient assessment*' (REN 4). The effort to redirect one's attention back to crucial care during interruptions was described as ‘*trying to deal with the interruptions and then returning back to main task and trying to stay focused. … Of course, I lose the thread*' (REN 1).

**Constantly receiving and processing information** was reported to be a strenuous cognitive activity. ‘*You get bombarded with so many things. It's just harder to think rationally and make the right choices because your brain has got so much going on at once….I feel like, scrambled wires just going everywhere and lighting up, like a highway*' (REN 5). The participant's metaphor of ‘scrambled wires' substantiated the internal pressure they were under, possibly due to cognitive resource depletion and excessive cognitive demand. Additionally, processing information amid distractions, triaging and obtaining vital clinical information were like ‘*cutting through a very long story to something that's more specific, more objective*' (REN 3). Discarding unnecessary information while selecting, categorising and storing vital information is effortful. ‘*You're just letting* [information] *go straight out your other ear because it's not important. And then you're having to … filter for the actual information you need at the same time… because you've already got enough in your brain*' (REN 9). These cognitive processes require intense attention necessary to filter and recognise the critical information under time pressure to prevent information clogging memory and other higher-order functions.

Most participants described their **excessive thinking** and **decision making**. They described constantly thinking and deciding on the priorities and effective strategies as a mental burden. ‘*You're always thinking, what's next?… I've gotta do this. I've gotta do that…Our brains are constantly going 1,000,000 miles an hour to the point that it is not as productive as it should be*' (REN 4). **Managing cognitive lists** of duties, responsibilities and tasks were raised. Being overloaded during work affected their performance as well as patient safety. ‘*When I have got so many things to do, there's no space in my mental thoughts*' (REN 2).

Often, they made **decisions under pressure** in urgency without sufficient time and taking into consideration all feasible options, and to ‘*miss small things*' evoked fear of making errors. ‘*In high-stress situations,… you just don't have the luxury of time to think through decisions and what you've done. That's when the mistakes do happen, because you just don't have the time to think*' (REN 8). Other participants echoed a similar sentiment of fear of mistakes when they were constricted by time while making crucial decisions. The elevated risk of inadvertent patient harm and acknowledgement of legal liabilities in the event of an adverse event created a sense of pressure. The internal pressure evoked self-blame, fear and anxiety. ‘*And what if they become really worse while they're waiting? And then it's obviously my responsibility. My fault for not getting to them quick enough*' (REN 5).

At the point of **cognitive saturation**, participants described experiences of cognitive failures like ‘brain fog' or ‘analysis paralysis', where a participant shared how this could impact on communication with other professionals. ‘*You definitely do get to a point where there's just too much to do; too many things to think. And even when you get to the point where if you call someone to help you; you probably can't even articulate what needs to be done*' (REN 6). REN 7 said, ‘*I think that humans only have a finite capacity to make a certain number of cognitively accurate, great decisions. And once you reach that threshold, you become overloaded with too many tasks on at once. As brain fog increases, your ability to make sound decisions, starts to deteriorate*' (REN 7). These experiences highlighted the risks that their decision making and performance could fail after reaching a particular threshold of high CMW.

All participants deployed **cognitive strategies** to deal with the chaos of ED, contain their CMW and deliver safe care. REN 9 stated that they reverted to a structured approach when they were faced with a challenge (patient deterioration). ‘*If anyone gets really sick, you just filter back to ABCD [airway, breathing, circulation, deficit-primary assessment]*' (REN 9). Utilising refocusing, REN 4 said, ‘*I remind myself what my role is at that point in time and remind myself what I'm doing*'. Hypervigilance to prevent errors included ‘*double checking*' (REN 2) and detect deterioration by ‘*watching for changes*' (REN 9). Additionally, there was evidence of nurses making time and resource-based decisions, such as ‘skipping' or not performing certain tasks or delegating tasks. The deliberate action of care rationing was to ensure that time was spent on the most important activities.

In summary, the participants' description of their experiences implies that inundation with information and task needs created high internal cognitive demand, requiring them to consume their limited cognitive resources. However, when the available cognitive resources become insufficient to meet the cognitive demand, participants experienced high CMW. The participants perceived the imbalance as feelings of pressure, having no space in brain, brain fog or feeling under attack by information and cognitive failures. Beyond the immediate experience of CMW, ‘*feeling like you are squeezed and if it kind of continues for a longer than few days, then definitely feel like changing my career choice*', demonstrated the risk of burnout and intention to leave profession among nurses.

### 5.3. Emotional Demand

Despite the chaos and heavy workload, the service-oriented nature of the participants' work demanded that they display empathy and compassion in every interaction with their colleagues, patients and the public. However, emotional suppression, faking calmness and emotional dissonance while delivering urgent care required intense mental effort. ‘*Anger does block some of your cognitive abilities and is occupied by anger, or there is this cloud of anger over your cognitive abilities*' (REN 1). The participants' mental energy investment in the form of cognitive resources generated emotional demand on top of their high cognitive demand, occupied their working memory and was associated with high CMW.

The challenge of **demonstrating compassion in a strenuous and challenging environment**, with the desire to alleviate suffering, was a common sentiment throughout the interviews. ‘*I don't want a patient in their final days to be in pain. That's just horrific*' (REN 5). Another participant stated, ‘*I just remind myself that no matter how busy we are, and how stressed we can be, this is still this patient's worst day. Most of the time that they're still here because they need help regardless of how busy we are*' (REN 4). All participants expressed genuine compassion and the importance of expressing it. However, because of high CMW, suppressing their emotions was effortful.

Most participants articulated an aching **desperation and guilt** resultant to difficulties in managing emotions ‘*Yes, you're trying to be compassionate with them, when you're talking to them and trying to explain to them what's going on. But sometimes you just have to go. Keep going without having a huge amount of time to explain and be very compassionate because you just got to do stuff to keep them alive*' (REN 5). Participants acknowledged that their care delivery without compassion display was not deliberate but rather was a necessity of performing lifesaving tasks and other priority clinical work that consumed them. Described as, ‘*feel torn between the two, but I'll prioritise the resus [resuscitation] patient and apologise to the palliative patient*' (REN 9). All participants prioritised urgent care over compassion, but their guilt and moral injury were an emotional cost. ‘*You might rush through changing their pad or taking them to the toilet or all that kind of stuff. And I think they feel less valued as a patient*' (REN 4). ‘*I feel frustrated and feel inadequate as a nurse that I can't give them the best that I want to give them*' *(REN 5).* This internal conflict felt like a moral injury, which added to their emotional demands.

Participants' ego depletion or decreased ability to regulate emotions was associated with **emotional suppression**. ‘*The ability to exercise, self-control and kindness reduces. You try and just push those emotions down and then just get on with the task. But sometimes the load gets so high that you can't push it down anymore*' (REN 7). The commitment towards preserving self-control while displaying kind emotions and maintaining a calm demeanour was a sentiment shared by all participants. Therefore, **modifying and strenuously controlling emotions** was evident. Most participants reported that faking emotions and hiding their genuine feelings required immense mental effort. When asked to describe ease to stay polite while dealing with disgruntled behaviours at work, ‘*I guess sometimes you just have to swallow the bitter pill and just take it on the cheek*' (REN 5). The incongruency in their actual and displayed emotions may have resulted in internal conflict leading to emotional dissonance.

But **creating a perception of calmness** was important: ‘*Calm outside, while churning inside*' (REN 1). REN 5 described faking as ‘a poker face', ‘*I think many years of doing the job (nursing), you sort of learn to have that poker face. I guess be calm on the outside but stressed to the max on the inside. But you know there can be a lot behind the poker face going on in my head*'. Furthermore, projecting calmness was used to prevent emotional contagion. ‘*I can be cool, calm, collected and confident, it projects that everything is under control. If patients get anxious,* that is *a whole other kettle of fish*' (REN 10). The statement illuminates the mental effort invested by REN 1 to stay calm and may have contributed to their high cognitive demand. ‘*I have like hundred tasks to do, and I have lined them up in my memory. I say to myself, It's not gonna work faster if you start panicking here. Just take a deep breath. […] so I basically, consciously perform some mental exercises to calm me down*' (REN 1).

Patients' expectations and nurses' expectations for themselves may lead to **moral injury, exhaustion and possible burnout**. Patients**'** sense of urgency raised conflict, ‘*the crowd refuse to understand that there's someone who's sicker, who needs my attention first*' (REN 8). ‘*I think that's what leads to the exhaustion… If you can't provide* [care] *because of the pressures of the shift, then I feel some moral injury from that, and I go home and reflect on that…. because I just feel like I haven't provided adequate care or the best care I could, on that day*' (REN 2). The need to maintain workflow was a **double-bind situation** and led to fear of reprimand, professional image degradation and potential litigation, adding to their emotional burden. Indeed, participants articulated their uneasiness about not meeting organisations' expectations. ‘*I am a lover of policy and procedure… However, due to high activity, high acuity, presentations, I had to work outside the rules. It makes me feel really uncomfortable, vulnerable and exposed*' (REN 10). Thus, there may be guilt and regret for taking shortcuts, even though it was a practical strategy to complete critical tasks within a limited time.

**Self-reproach, overthinking and catastrophising** were also evident among most participants. ‘*Sometimes I feel that the resuscitation of patients was messy and not 100% satisfactory. I believe we could have done better. This often leads me to self-criticism*' (REN 1). Also, overthinking and catastrophising after work generate unease about work. ‘*The things that stressed me out the most were the after-effects. Was everyone OK? Did I give the right category [triage category]? Did anyone deteriorate in the waiting room? Just all those kinds of thoughts, which then added on to my fatigue (after work).* That is *where most of my compassion fatigue kind of came from*' (REN 9).

In summary, emotional demands resultant from emotional management occupied working memory and depleted cognitive resources needed for other crucial cognitive processes. The resource depletion created a scarcity under high cognitive demand and resulted in their experience of CMW.

### 5.4. Individual Characteristics

Surviving in a ‘chaotic' and ‘stressful' environment under high cognitive and emotional demands requires personal traits and qualities such as stress tolerance, conscientiousness, cognitive agility and expertise.

**Stress tolerance and conscientiousness** were necessary for attention to duty of care. This means ‘*multitasking and having to deal with the stress and still trying to do a good job*' (REN 5). Participants expressed their inner dispositions to push forward with their care task even under adverse conditions. ‘*Even when really busy with high cognitive workload, you're still doing these tasks*' *(REN 7).* An ability to tolerate excessive stimulation was another trait. ‘*People who work in ED generally like that little bit of stimulation, that kind of higher level of stimulation, and maybe that's what filters people out early in their career…. However, when I get to a point where it's been stressful, I do have that moment, and think, Can I do it again tomorrow?*' (REN 2). ‘*…but then if you have to do it all the time, it gets exhausting*' (REN 8). This ostensible high stress tolerance seemed to be an essential trait for an emergency nurse's high CMW endurance.

**Cognitive agility** is the mental ability to rapidly switch and adapt thinking, action and behaviour to unpredictable changes in ED. An example was, ‘*You never know what's gonna come through the doors. Sometimes, you might be just chilling there, reading a book or doing mandatory training. Next minute, you just sort of have to snap into that gear and actually just really get in that work*' (REN 8). The rapidity at which the nurses could switch into action required sudden thought, behaviour and action change, which proves their cognitive agility. Their agility enables swift assessment of the situation, rapid information processing, thinking and problem solving to provide care with their limited available resources.

Participants confirmed that toleration of stimulation and chaos improved with **experience and skill acquisition**. ‘*I've been doing nursing for a while now, but I don't know how younger or novice practitioners filter it (stimulation in ED) out*' (REN 2). Thus, skills improved with years of exposure and experience. REN 10 reported repeated exposure and performing the same skills reduced mental effort in executing the task, ‘*In my career, so far, I've done a lot of these patients (with chest pain)…. So, it's kind of is a bit of that unconscious competence where you just go on autopilot. And I know all the things I have to do, so I just go and do them*'. This expertise reduces mental effort in assessing, planning and executing tasks for a common condition. REN 3 said, ‘*I think the more experienced you are, your cognitive load would come down. Because I certainly feel that I'm a very different nurse than I was four years ago in ED, and that would be primarily due to the experiences that I've had in those four years*'.

In summary, the participants' endurance, perception and ability to cope with CMW were mediated by their individual traits, abilities, knowledge, exposure and expertise to manage stress. That is, individual characteristics of participants interacted and influenced all three other components of CMW and cognitive resource utilisation.

#### 5.4.1. Difficulty in Voicing Unpleasantness

All participants were in consensus, affirming that ‘*the cognitive load is quite high*' (REN 7). With 10 years of experience in ED, REN 2 explained the phenomenon as a **balancing act** of multiple cognitive processes; ‘*cognitive workload feels like juggling many balls in the air, with constant demands from patients, staff, and random interactions. It involves continuous decision-making and processing numerous inputs, making it challenging to manage everything at once*' (REN 2). While for a few, it was **the external pressure** they perceived during their work: ‘*Once you're at that level of high cognitive workload (CMW), anything on top of* that is *just like the straw that broke the camel's back*' (REN 6). REN 2 also attempted to describe CMW using their **distressing feeling**, ‘*You're like a rat on a ring and you're burning yourself and burning through your adrenaline*' (REN 2). Other participants related CMW to **unpleasantness** during their highly stressful work in high stake situations. For REN 1, CMW was ‘*things coming at me*'. REN 3 defined it as, ‘*doing awful lot of things at the same time and constantly triaging in head what to do next*'. Another participant described CMW as ‘too *much pressure on brain and too many different avenues requiring attention at one time that you just can't totally process each thing properly*' *(REN 4)*. Thus, different individuals perceived the phenomenon differently.

On the contrary, a few participants admitted enjoying certain levels of CMW and highlighted it as essential for achieving the best performance. ‘*If you're not stimulated, if you're not alert at all, then you're likely not to do things and not even realise it*' (REN 6). REN 7 appreciated CMW until it reached a certain level beyond which it became bothersome. ‘*I like the feeling to the point of it (CMW), challenges me. It makes me work hard. It makes me better as a nurse. Uhm, to have me critically think and come up with solutions. But I don't like it when I am put in a position where patient safety is at risk*' *(REN 7).* Thus, both low and high CMWs are challenging during care delivery.

In summary, it can be inferred from the interview data that the participants' optimum CMW is between the hypothetical juncture at which their performance was simulated to the point at which it affected patient safety. ‘*I like the feeling to the point of it (CMW), challenges me. It makes me work hard. It makes me better as a nurse. Uhm, to have me critically think and come up with solutions. But I don't like it when I am put in a position where patient safety is at risk*' (REN 7). However, both high and low points of optimal CMW seemed arbitrary and are yet to be established.

## 6. Discussion

This research aimed to understand the lived CMW experiences through the voices of emergency nurses. Ten RENs were interviewed to collect data analysed using RTA. This discussion outlines the key findings in relation to external demands, emotional demands, cognitive processes and individual characteristics.

The main finding of the study was that the nurses' CMW experiences emanated from cognitive resource depletion caused by emotional demand and excessive cognitive processing in response to external demands (environmental and work characteristics). These experiences may be influenced by individual characteristics (depicted in Figure 1). The finding is partially congruent with cognitive psychology, ergonomics and human factor theories [9, 10]. That is, high CMW is driven by excessive cognitive resources necessary to manage cognitive demands felt in response to challenging environments and work. The pervasive negative commentary from all participants was consistent with previous research [3, 11], indicating chronically high levels of emergency nurses' CMW. However, difficulties in precisely articulating their experiences of the phenomenon and the vague descriptions of optimum CMW level may have affected their ability to seek help to change their work conditions.

### 6.1. Emotional Demands

Participants' emotional demands were impacted by the need to express socially and professionally acceptable displays of emotion, which significantly contributed to their high CMW. The finding contradicts Van Acker et al. [10], who argued that emotional load is merely a situational appraisal and not a causative factor of CMW; therefore, it must not be included as a defining aspect of the phenomenon. Most participants openly acknowledged the mental effort required to manage challenging patient interactions with compassion and professionalism while maintaining self-control. Aligning with Kirk et al.'s [4] finding, participants suppressed, concealed and or modulated their emotions to display professionally acceptable behaviour.

Allocating limited working memory resources increased their cognitive demand and cognitive resources depletion for emotional management, which led to resource scarcity [10]. The imbalance between available resources and cognitive demand likely contributed to participants' CMW. Unpleasant feelings of CMW could be the reason why participants reported anxiety, moral injury and self-reproach, leading to companion fatigue, burnout and intention to leave the profession. This finding is similar to Davids et al.'s [26] well-being study in Australian EDs. Therefore, addressing the emotional and social welfare of emergency nurses through targeted support and zero tolerance for aggression is essential for CMW optimisation, mitigating compassion fatigue and sustaining workforce resilience.

### 6.2. Cognitive Processes in Response to External Demand

Participants reported high CMW experience while performing various cognitive processes like maintaining vigilance, information processing, thinking and decision making in excess during emergency or workflow crisis under time pressure. This finding made sense when we analysed the data with attention resource theory [27], information processing theory [28] and cognitive load theory [6]. Warm et al. [27] suggested that maintaining vigilance or sustained attention is mentally demanding, during which cognitive resources get replenished more quickly than it can be restored in human cognitive architecture. That is, when the participants were being vigilant to critical events and cues in their environment, they experienced high CMW due to excessive cognitive resource expenditure. Information processing theory [28] provides insight into how participants collected, sorted, encoded and recalled information in their limited working memory by utilising cognitive resources. Cognitive load theory posits that human working memory has a limited capacity, and when cognitive demands exceed this capacity, cognitve processing are impaired, resulting in delay or decline in performance and human error [6]. That is when participants were performing excessive information processing, thinking and decision making, it was beyond their limited working memory capacity, with scarcity of resources, that led to their CMW experiences and its outcome.

The theories facilitated understanding of why participants commonly described their CMW as ‘high pressure' and ‘no space' in their brains. Dean et al. found that mothers experienced pressure related to keeping their children safe, securing their future, and balancing work and family responsibilities as part of their CMW [29]. Their disclosure of ‘high pressure' was often related to their intangible cognitive process, like excessive information processing, constant thoughts and excessive decision making for task execution, meeting patient needs and responsibilities, possibly resulting in internal pressure. The participants' ‘no brain space' articulation in such circumstances may be due to the complete utilisation of their limited human working memory capacity with multiple cognitive functions. On the other hand, external pressures were induced by time pressure, urgency, work complexity, intensity and work abundance. Therefore, anything in surplus in their internal (viz. information processing, thinking, decision making, cognitive tasks and problem solving) and external (viz. stimuli, distractors, interruptions, waiting patients, time pressure and complexity) environment leads to their experience of CMW. This finding is consistent with previous research, where excessive cognitive process overwhelms limited working memory capacity, depleting limited cognitive resources, resulting in imbalance [9, 10]. Often, the state resulted in an inability to function any further, leading to a state of decision fatigue, a cognitive process decline designated as ‘brain fog' by participants. The finding aligns with a recent qualitative study of frontline nurses that linked decision fatigue to workload [30]. It could also be the reason why participants linked their CMW with error, missed care and adverse events, as well as their fatigue, exhaustion and burnout which is similar to Gündüz and Öztürk's [17] descriptive cross-sectional study of 156 Turkish ICU nurses.

Participants described CMW with workflow disruptions such as frequent task switching, interruptions and distractions. The nurses' apparent lack of agency during workflow turbulence, possibly due to high CMW, contributed to delays or missed critical cues, performance lapses, forgetfulness and heightened risk of error, therefore reinforcing participants' feelings of powerlessness, fears of mistakes and feelings of incapacity to deliver care. Parallel to Jennings et al. [31], the findings underscored the critical link between workflow disturbances and their well-being.

Often, to safeguard patient safety and care quality, participants conscientiously maintained their high performance despite enduring intense CMW, recognising that any error could result in consequences ranging from minor discomfort to fatality, even when the intense mental effort negatively impacted their welfare. Their coping capacity was noted to be like the concept of endurance [32]. Endurance is sustaining performance and safety in extreme work environments (viz. spaceships and arctic expeditions) owing to high failure cost, further depleting participants' cognitive resources and intensifying their CMW.

### 6.3. Individual Characteristics

In this study, the emergency nurses' perceived CMW intensity and endurance capacity varied with individual traits such as cognitive agility, stress tolerance, conscientiousness, experience, exposure and emotional state. Al-Moteri [33] found that while emergency nurses received identical patient information in simulated scenarios, their cognitive processes, such as information processing, thought processing and decision making, varied significantly across individuals. The variations in cognitive processing with Guastello et al.'s [18] theory of the perceived CMW intensity and endurance difference with individual traits such as stress tolerance, conscientiousness, experience, exposure and emotional state support participants' reported differences in CMW perception and experience. This also explains why some participants described how enduring and coping with CMW was a learned ability that improved with experience and exposure.

### 6.4. Optimum CMW

Despite the diversity in perceptions, optimum CMW was discussed in terms of high and low CMW at which participants' performance was ideal. However, participants were unclear about the threshold that defined these levels. They used indicators such as the point at which CMW affected patient safety and performance to describe their optimal CMW, but this was inconclusive. This finding raises the question: At what level does CMW begin to impact patient safety and nurses' performance? In other words, what is the threshold at which emergency nurses can provide quality patient care and perform optimally?

Furthermore, multifaceted, intangible and implicit nature of CMW made articulation and quantification challenging. Participants articulated their experience of the phenomenon through emotional descriptors (viz. frustration, overwhelming and pressure), demanding cognitive processes, challenging circumstances and adverse clinical outcomes. Observable indicators like error incidents, adverse events and their own performance delays or failures were also used to express the phenomenon. Participants frequently linked ‘juggling' and ‘balancing' of multiple stimuli, excessive thoughts and multiple decision making in their interruption prone environment of ED to their CMW. Additionally, they considered multitasking and task stitching as high CMW. For a few participants, high CMW was the feeling of situations getting out of hand or losing control of the situation around them, the associated fear and anxiety of making mistakes. Similar to Eriksson et al.'s [34] study, a few participants expressed feelings of overburden and loss of control feelings resulting in overcrowding and access blocks, as CMW, which resulted in a feeling of being inadequate as a nurse owing to the inability to meet patients' basic needs and protect them from errors. However, the rarity of CMW qualitative research limited the comparison of meanings attributed by emergency nurses to the general population.

Congruent with the articulation difficulties, the prior established nonexistence of customised subjective modalities to measure emergency nurses' CMW [2] complicated the understanding of the phenomenon. The dearth of research into CMW may have contributed to limited optimisation strategies, lack of awareness and policy interest in the area.

### 6.5. Strengths and Limitations of the Work

This pioneer study, with its data richness and comprehensiveness, succeeded in offering a framework for future CMW studies among nurses and other health professionals. To our knowledge, this is the first known study that has explored the lived CMW experience of nurses during their care delivery, and with nurses located from various rural locations across Australia.

A key strength of this study was the integration of *insider* (AS) and *outsider* (PM and LB) perspectives within RTA, which enabled a rich, nuanced approach to meaning making. The insider contributed contextual sensitivity and cultural insight, while the outsiders provided critical distance and challenged assumptions, fostering reflexive dialogue that enhanced interpretive depth and transparency. The balance strengthened analytical rigour and trustworthiness, which is consistent with Big-Q qualitative paradigms that view subjectivity as a resource rather than a bias. Additionally, the study employed contemporary methodological innovations, including the use of information power [20] to guide sample size decisions and the BQQRG framework for reporting [19]. Both align with the epistemological foundations of RTA. Furthermore, the study protocol was preregistered to improve transparency.

There are limitations to note. As purposeful sampling was necessary to ensure data consistency to achieve information power, a single-country study may have prevented capturing experience variations across cultures, nurses' education and training and organisational management systems. Although the homogeneity of the sample provided in-depth knowledge of the phenomenon, it concomitantly reduced the generalisability of the findings. Additionally, voluntary participation, online consenting and digital interviews introduced selection bias because participants with basic digital skills and internet access could not join the study. The use of online audio recording of interviews also restricted access to nonverbal cues and contextual observations. These limitations are not insurmountable for future research.

### 6.6. Recommendations for Further Research

1. The utilisation of multiple descriptors to express their CMW proves the complexity and multidimensional nature of the phenomena. That could be the reason for not seeking system change by emergency nurses even when enduring an elevated level of CMW. Therefore, replicating the research in various settings, multisite, cultures and countries, as well as among diverse groups of nurses and other health professionals, can broaden our understanding of the phenomena.
2. Emergency nurses' CMW framework, depicted in Figure 1, lays the groundwork for further research into nurses' CMW optimisation. Further research is essential to refine and establish the interactions and links between the components in the framework. Similar studies in various nursing roles, other health professions, various settings and other countries in varying methodologies could provide a deeper understanding of the phenomenon, which can inform CMW optimisation and implementation of error prevention strategies.
3. Emotional demand preceded participants' CMW experience and was not always a resultant appraisal of high CMW in participants. The finding diverges from the dominant perspective and calls for additional research to determine the direction of effects.
4. Currently, there are no specific subjective measures of CMW for emergency nurses [2]. Therefore, the development of a customised subjective measure with insight from this study could facilitate the phenomenon's quantification. Such a scale and CMW quantification may inform CMW optimisation to safeguard the welfare of both patients and nurses.

### 6.7. Policy Implications

1. Ensuring optimal CWM levels for nurses is essential for safe, high-quality urgent care and reducing staff turnover costs resulting from burnout and patient harm–related expenditures. Additionally, the critical link between turbulent workflow and participants' CMW highlights the need to design emergency nurses' work environments to minimise turbulence and reduce CMW. Therefore, we urge policymakers to consider insight from the study during healthcare planning and policymaking to enhance the well-being of patients and nurses.
2. The complexity of CMW and difficulty in its articulation may contribute to its normalisation in workplace cultures and hinder calls for system changes. Policy should support clearer recognition and responsive action to address CMW in emergency settings and in other patient care settings to avoid patient harm resulting from human error and reduce staff turnover caused by burnout.

### 6.8. Practice Implications

1. Emotional demand was a major contributor to participants' CMW experiences. Instead of emotions developing in response to CMW [10], these participants' emotional demand preexisted and contributed to high CMW. That is, providing sufficient emotional and psychological support as well as emotional offloading through structured daily debriefing and specialised services after major events must be considered to ensure emergency nurses' well-being.
2. Certain personal traits help nurses cope with and endure CMW. Identifying these traits and using this knowledge in job suitability testing can help emergency nursing managers select appropriate nurses for their ED.
3. Acknowledging participants' remarks that enduring and coping with high CMW is a learned skill, CMW knowledge and management strategies should be integrated into the nursing curriculum and induction programs, like what is provided during surgical training [35].

## 7. Conclusion

Overall, the study offered new insights into emergency nurses' complex, multifaceted experiences of CMW. The study found that emergency nurses' persistently high CMW experiences impact their well-being and patient safety. Key contributing factors of high CMW were external demand, emotional demand and cognitive processes, along with individual characteristics, shaping their perceptions, appraisals of the demands, endurance and coping abilities. This study highlights the need for work system changes to optimise CMW and enhance workflow, emphasising the importance of addressing cognitive demands and emotional pressures to improve patient safety, reduce human error and mitigate burnout, ultimately benefiting the healthcare sector. In summary, the study insights are crucial for work system planning and CMW optimisation strategies for safe patient care delivery and sustainable healthcare workforce welfare.

## Figures and Tables

**Table 1:** Braun and Clarke's RTA process application in the current study.

RTA phases	Actions, approaches and outcomes
Phase 1: Familiarisation	Active listening of audio recording of each participant Transcript of each recording was read and reread multiple times to engage with the data Cross checking of transcript with audio recordings Reflexive journalling by writing memos in NVivo
Phase 2: Generating codes	Each line manually coded to identify as well as attributing meanings to simplest unit of data Both semantic information and latent information were coded Reflexive journalling by writing memos in NVivo Team member checking
Phase 3: Generating themes	Patterns were identified and codes were organised based on a specific commonality Thematic mapping in PowerPoint resulting in active and organic emergence of themes and subthemes Reflexive journalling by writing memos in NVivo Team member checking
Phase 4: Developing and reviewing themes	Total 290 codes were identified. The codes were divided among 58 emerged themes and 184 subthemes The themes and subthemes were then categorised into seven clusters based on contribution to phenomena under study. Owing to large number of themes and subthemes, decision was made to analyse only one of 7 clusters that provided direct information about the nurses' lived CMW experiences, by adapting ‘partial data set analysis' technique proposed by Braun and Clarke ([22], p. 101) Reflexive journalling and team member checking performed
Phase 5: Defining and naming themes	Emerged themes and subthemes in cluster 4 were merged, divided and or omitted to make sense of data through analysis and team member checking Naming and defining of 10 themes with 61 subthemes in cluster 4 were carried out to demarcate each theme/subtheme concept margins Thematic mapping followed by team member checking performed to define and understand relationship between subthemes
Phase 6: Write up	Analytical writing and member checking of 10 themes and 61 subthemes were carried out. Selection of excerpt to construct story board [23] for answering research question was performed Write up performed to iteratively demonstrate how the analysis addressed the research question

## Data Availability

The datasets generated and analysed during this study are not publicly available to safeguard their use in planned future research. Access may be granted upon reasonable request to the corresponding author.
